# Analyzing Obesity Trends in American Children and Adolescents: Comprehensive Examination Using the National Center for Health Statistics (NCHS) Database

**DOI:** 10.7759/cureus.61825

**Published:** 2024-06-06

**Authors:** Oluwatosin B Iyun, Okelue E Okobi, Elochukwu U Nwachukwu, Wendy Miranda, Natalie O Osemwegie, Roseline Igbadumhe, Adedoyin Olawoye, Chika C Oragui, Nnenna A Osagwu

**Affiliations:** 1 School of Public Health and Family Medicine, University of Cape Town, Cape Town, ZAF; 2 Family Medicine, Larkin Community Hospital Palm Springs Campus, Miami, USA; 3 Family Medicine, Medficient Health Systems, Laurel, USA; 4 Family Medicine, Lakeside Medical Center, Belle Glade, USA; 5 Family Medicine, University of Uyo Teaching Hospital, Uyo, NGA; 6 General Surgery, University of Ghana Medical Center, Accra, GHA; 7 Medicine and Surgery, Igbinedion University, Benin, NGA; 8 Psychology, Alaska Native Medical Center, Anchorage, USA; 9 Internal Medicine, Maimonides Medical Center, New York, USA; 10 Pediatrics/Pediatric Intensive Care Unit, Stanford University School of Medicine, Lucile Packard Children's Hospital, Palo Alto, USA; 11 Department of Medicine, All Saints University School of Medicine, Roseau, DMA

**Keywords:** national center for health statistics (nchs), trends, adolescent, children, obesity

## Abstract

Background

In the USA, obesity in children and adolescents has become a major public health concern. Childhood obesity has been linked to various cardiometabolic comorbidities all through one’s life. Owing to the significant increment in childhood obesity rates, there has been an urgent need for the identification of the correlates and antecedents of adiposity and the cardiometabolic risk to enable early prevention of obesity. As such, the objective of this study is to analyze obesity trends in American children and adolescents from 1999 to 2018 using the National Center for Health Statistics (NCHS) database, as this will enable the identification of various risk factors and early prevention of childhood obesity.

Objective

This study aimed to comprehensively examine demographic factors impacting obesity prevalence, including gender, age groups (two to five, six to 11, and 12-19 years), race/ethnicity, and poverty level.

Methods

The study conducted a retrospective analysis using the NCHS database from 1999 to 2018. Utilizing NCHS data, we examined the evolution of obesity prevalence among children and adolescents. The analysis focused on demographic variations, including gender, age, race/ethnicity, and percentage of poverty level. SPSS version 24, a statistical software by IBM Corp. (Chicago, IL, USA), was used for database summarization, graphical representation, and presenting prevalence trends across all participants.

Results

Temporal trends in obesity prevalence exhibited notable fluctuations from 1999 to 2018. Utilizing NCHS data, the study revealed demographic disparities in age groups, genders, race/ethnicities, and socioeconomic status categories. Gender-based obesity variations persist, with boys consistently surpassing girls in prevalence (17.5% vs. 16%, p = 0.0231). Varied age group patterns emerged, peaking at 18.7% in 12-19 years, 17.7% in six to 11 years, and 11.2% in two to five years. Racially, Hispanic individuals had the highest prevalence (22.8%), followed by Mexican (22.0%) and Black or African American-only individuals (20.6%). White-only individuals showed 14.4%, and Asian-only individuals exhibited the lowest (9.4%). Lower socioeconomic brackets correlate with higher obesity instances, particularly below the 100% poverty level (20%). The 100-199%, 200-399%, and 400% or more categories contributed 18.6%, 16.6%, and 11.6%, respectively.

Conclusion

Our extensive examination of obesity trends among American children and adolescents from 1999 to 2018, utilizing the NCHS database, provides valuable insights into the complex interplay of demographic factors influencing this public health concern. The study reveals age-specific variations, emphasizing unique challenges during adolescence. Gender disparities, socioeconomic influences, and racial/ethnic impacts are evident, underscoring the need for further study. Our findings present several policy implications regarding the development of interventions aimed at reducing childhood obesity rates in the USA. For instance, the findings indicate the need for policymakers to develop policy interventions aimed at enabling the prevention of obesity during early infancy stages. The findings highlight the need for interventions aimed at reducing the obesity disparities observed between genders and races/ethnic groups. Developing and executing the interventions is prone to considerably reduce the obesity prevalence rates among children and adolescents in the USA.

## Introduction

Obesity, recognized as a global health concern, mainly results from the imbalance between energy intake and expenditure, which, in turn, leads to the excessive accumulation of body fat. Obesity has also been acknowledged to be caused by genetics, certain medications, and various underlying health conditions. Its prevalence has surged, driven by sedentary lifestyles, unhealthy dietary habits, and genetic predispositions [1]. Obesity poses substantial health risks, contributing to the development of chronic conditions such as diabetes, cardiovascular diseases, and certain cancers. In addition, it impacts mental well-being, exacerbating societal health burdens. Addressing this complex issue requires comprehensive strategies, emphasizing education, lifestyle modifications, and supportive environments to foster healthier choices and curb the escalating global obesity epidemic [2].

The World Health Organization (WHO) defines obesity and overweight in children and adolescents based on the body mass index (BMI) for age. Obesity is characterized by a BMI-for-age greater than or equal to the 95th percentile on the WHO growth standards, while overweight falls between the 85th and 94th percentiles. These classifications consider age and gender variations in growth patterns. Monitoring the BMI in children and adolescents is crucial for identifying and addressing potential health risks associated with excess body weight, promoting early intervention, and encouraging healthy lifestyle habits [3-4]. Moreover, in children and young adults, the waist circumference measurement has been used as the most appropriate determinant of central obesity. Moreover, waist circumference has been acknowledged as a better marker for central obesity development, in addition to being a better predictor of cardiometabolic risks in adolescents and young adults. Thus, in adolescents and young adults, waist circumferences of over 94 centimeters in males and above 80 centimeters in females are indicative of central obesity [3-6]. Moreover, a waist circumference percentile of ≥95th has additionally been acknowledged to indicate the existence of central obesity in children and adolescents [3-6].

In children and adolescents, obesity can result from a complex interplay of genetic, environmental, and behavioral factors. The condition poses significant health risks, including the development of chronic diseases, such as diabetes and cardiovascular issues. Over the past few decades, childhood obesity has emerged as a persistent issue in the United States, with rates tripling since the 1970s. In 2023, the prevalence remained alarming, with one in five American children classified as obese, and this number continues to escalate annually [5]. Moreover, in the demographic spanning children and adolescents aged two to 19 years during the period from 2017 to 2020, obesity emerged as a significant public health concern, impacting approximately 14.7 million individuals with a prevalence of 19.7%. Delving into specific age groups, the prevalence varied, with 12.7% among two- to five-year-olds, 20.7% among six- to 11-year-olds, and 22.2% among 12- to 19-year-olds [6].

In children and adolescents, obesity manifests through complex pathophysiological mechanisms. Excessive calorie intake, sedentary behaviors, and genetic factors contribute to an imbalance in energy regulation. Adipose tissue, particularly visceral fat, undergoes hypertrophy, leading to an inflammatory state and increased secretion of adipokines [7]. Insulin resistance often ensues, disrupting metabolic homeostasis [8]. Dysregulation of appetite-regulating hormones and altered gut microbiota further exacerbate the condition [9]. These interconnected processes contribute to chronic low-grade inflammation, insulin resistance, and metabolic dysfunction, collectively underpinning the pathophysiology of obesity in children. This emphasizes the importance of multifaceted interventions for effective prevention and management [10].

The National Center for Health Statistics (NCHS) database stands as a cornerstone in healthcare research, providing a robust repository of comprehensive health-related data. Its expansive datasets, spanning diverse demographics and health indicators, enable comprehensive analysis and evidence-based insights. The NCHS database plays a pivotal role in shaping public health discourse, offering a rich resource for researchers and policymakers to understand trends, inform strategies, and enhance the overall well-being of populations [11]. Therefore, the primary objective of this study is to derive insights from the NCHS database, providing a comprehensive understanding of the factors contributing to obesity prevalence in this demographic. This exploration of the dataset aims to address the multifaceted challenges posed by childhood and adolescent obesity in the country.

## Materials and methods

Data source and study design

A retrospective analysis was conducted using data extracted from the NCHS database, covering the substantial period from 1999 to 2018. Acknowledged as a pivotal and authoritative repository of health-related information in the United States, the NCHS database played a crucial role as the primary data source for this investigation. It offers a comprehensive collection of demographic, health, and nutrition data obtained through various surveys, including the National Health and Nutrition Examination Survey (NHANES) [12].

Study participants and inclusion and exclusion criteria

Given that this study focuses on obesity in children and adolescents, the definition of obesity has been based on sex/gender and age definite normograms for BMI. In this regard, for this study, obese was defined as children or adolescents with BMIs equivalent to or surpassing the age/gender definite 95th percentile, even as those with BMI equivalent to or surpassing 85th percentile but below 95th percentile have been defined as overweight and at higher risk for obesity and associated comorbidities. As such, the inclusion criteria comprised children and adolescents aged between two and 19 years and selected to comprehensively cover the developmental stages of childhood and adolescence. The study included individuals who participated in relevant surveys conducted by the NCHS during the study period, spanning from 1999 to 2018. The exclusion criteria included individuals aged below two years and those aged above 19 years. Furthermore, individuals who presented conditions other than obesity. Lastly, the exclusion criteria included individuals who participated in pertinent surveys conducted by the NCHS before 1999 and after 2018. 

Data collection and quality assurance

Relevant datasets were systematically retrieved from the NCHS database using standardized protocols. Rigorous attention was given to data quality and consistency, ensuring precision in the analysis. Data cleaning procedures involved addressing missing values, outliers, and inconsistencies. To handle the missing data, some of the observations with missing values were dropped, even as those with more accurate observations had their missing values inserted based on the integrity and accuracy of the observations. Consequently, the inconsistencies were addressed by removing the unwanted observations from the datasets, including irrelevant and duplicate observations. The dataset was strategically aggregated by year, age groups, gender, and other pertinent categories to facilitate a thorough and structured statistical examination.

Variables of interest

The primary focus of the study was on obesity patterns, with relevant indicators and classifications obtained from the NCHS database. Demographic attributes, including age, gender, and race/ethnicity, were collected to assess potential correlations with obesity prevalence. Age groups were categorized into two to 19 years to encompass the entire range of childhood and adolescence. Gender was recorded as male or female, while race/ethnicity included categories, such as Hispanic or Latino, Mexican, Black or African American-only, White-only, and Asian-only. In additon, socioeconomic indicators, specifically the percentage of the poverty level, were incorporated to provide insights into the potential influence of economic factors on obesity rates. Poverty levels were categorized into four groups: below 100%, 100-199%, 200-399%, and 400% or more. Temporal variables were considered to identify trends and patterns in obesity rates over different years. The above variables were selected for several reasons including the observation that the variables would enable policy-makers to develop policies and educational information targeting the most affected population demographic. Thus, this will enable the development of customized policies for each group, thereby enabling the effective reduction of obesity rates in the United States.

Data analysis and statistical methods

The NCHS datasets underwent rigorous cleaning and preprocessing to ensure data integrity. Careful consideration was given to missing values, outliers, and inconsistencies. This phase involved data cleaning, rigorous validation checks, and, where required, imputation of missing values. The dataset was strategically aggregated to facilitate a thorough and structured statistical examination by year, age groups, gender, and other relevant categories. Consequently, the statistical analyses were conducted using SPSS version 24, a statistical software program developed by IBM Corp. (Chicago, IL, USA). Thus, a one-way analysis of variance (ANOVA) was utilized in the examination of variations in obesity rates across different demographic attributes (age, gender, and race/ethnicity) and socioeconomic indicators (percentage of the poverty level). ANOVA was chosen for its suitability in assessing mean differences among multiple groups simultaneously. A significance level of 0.05 was employed to determine statistical significance. If the p-value from the ANOVA test was less than 0.05, it indicated statistically significant differences in obesity prevalence among various demographic and socioeconomic categories.

Ethical considerations

This study utilized de-identified, publicly available data from the NCHS, ensuring the privacy and confidentiality of participants. No additional ethical approval was required for the analysis of secondary data [8-12]. All data handling procedures strictly adhered to the confidentiality standards outlined by the NCHS, with the research team having access only to anonymized and aggregated data, maintaining the utmost privacy of participants. These rigorous ethical considerations enhance the credibility and acceptability of the study for publication in scientific journals.

## Results

A comprehensive analysis of two decades of NCHS data has provided valuable insights into the prevalence, demographic variations, and temporal shifts in obesity among children and adolescents. Spanning from 1999 to 2018, this extensive study delved into the patterns of obesity prevalence within this demographic. The subsequent presentation of results in Table 1 offers a detailed overview of key findings, illuminating the role of selected characteristics in shaping obesity patterns among American youth.

**Table 1:** Obesity among children and adolescents aged two to 19 years by selected characteristics - Not available. * p-value (<0.05*) represents a significant value.

Variables		1999–2002	2001–2004	2003–2006	2005–2008	2007–2010	2009–2012	2011–2014	2013–2016	2015–2018	P value
Total data	Both sexes (2–19 years)	14.8	16.3	16.3	16.2	16.8	16.9	17.0	17.8	18.9	-
Data based on race	White only	12.6	15.1	14.7	14.1	14.6	14.0	14.7	14.7	15.1	< 0.05*
	Black or African American only	18.2	18.8	20.7	20.7	22.1	22.1	19.5	20.4	23.1	
	Asian only	-	-	-	-	-	-	8.6	9.8	9.8	
	Hispanic or Latino	-	-	-	-	21.1	21.8	21.9	23.6	25.7	
	Mexican origin	20.0	19.4	20.9	21.7	21.0	21.9	22.2	24.2	26.9	
Data based on gender	Boys	15.5	17.3	17.0	16.8	18.2	17.6	16.9	18.1	19.8	0.023*
	Girls	14.1	15.2	15.5	15.5	15.4	16.1	17.1	17.5	17.9	
Data based on poverty	Below 100%	17.6	17.9	18.9	19.9	20.8	20.9	19.4	21.0	23.9	< 0.05*
	100–199%	15.3	16.7	17.4	18.2	18.3	18.5	20.3	20.7	21.7	
	200–399%	14.0	17.8	17.1	16.0	16.7	15.9	16.4	16.9	18.4	
	400% or more	12.6	11.2	11.0	11.6	11.9	11.5	11.4	12.2	10.6	
Data based on age group	2-5 years	10.3	12.4	12.5	10.5	11.1	10.2	8.9	11.6	13.7	< 0.05*
	6-11 years	15.9	17.5	17.0	17.4	18.8	17.9	17.5	17.9	19.3	
	12-19 years	16.0	17.0	17.6	17.9	18.2	19.4	20.5	20.6	20.9	

Based on gender

The examination of obesity prevalence reveals significant gender-based variations. Across the study period from 1999 to 2018, a consistent trend emerges, indicating that boys consistently exhibit a higher prevalence of obesity compared to girls. Nevertheless, during the years 2011-2014, a temporal proximity was observed where the obesity percentage for boys was 16.9%, slightly lower than that for girls, which was 17.1%. The observed trend (2011-2014) can be attributed to a number of factors that include the sedentary lifestyle of female children and adolescents in comparison to their male counterparts who partake in various physical activities. Nevertheless, for the overall study duration (1999 to 2018), boys accounted for approximately 17.5% of cases, while girls comprised 16% of the cases of obesity.

**Figure 1:**
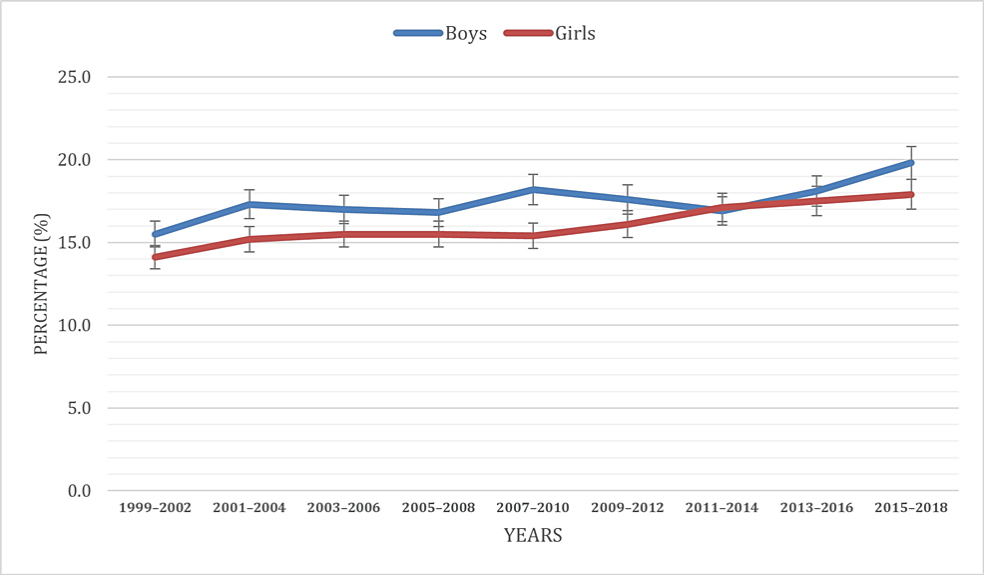
Obesity prevalence based on gender

Statistical analysis indicates that the observed differences in obesity prevalence between boys and girls are statistically significant (p = 0.0231), suggesting that gender-based variations are not likely due to random chance. These findings emphasize the importance of considering gender-specific factors in understanding and addressing obesity trends over time. Further temporal analysis elucidates these disparities. For girls, there was a steady increase in obesity prevalence across the study period, with a slight decline of 0.1% observed from 2006 to 2010. By contrast, the trend for boys exhibited an initial rise from 1999 to 2004, followed by a decline from 2004 to 2008, stabilizing around 17.3% to 16.8%. From 2008 to 2010, there was an increase from 16.8% to 18.2%, followed by a decline from 18.2% to 16.9% from 2010 to 2014. Subsequently, from 2011 to 2018, there was another increase from 16.9% to 19.8%. These observations align with prior research findings and underscore the importance of considering gender-specific factors in the prevalence of obesity among children and adolescents aged two to 19. Figure 1 visually illustrates the gender distribution of obesity cases among children and adolescents throughout the study period. 

Based on the age of children

A comprehensive examination of age-specific obesity prevalence offers insightful perspectives into the manifestation of this public health concern across distinct developmental stages among American children and adolescents. Our analysis, conducted through an ANOVA test, revealed significant variations in obesity rates across different age groups (p < 0.001) and discerned variations in obesity rates across different age groups, elucidating distinctive patterns within the two to 19 years range. The statistical analysis underscores the robustness of these age-specific differences in obesity rates. Furthermore, the highest prevalence was observed in the 12-19 years age group (18.7%), followed by the six to 11 years group (17.7%), and the two to five years group (11.2%).

Temporal trends unveiled the dynamic landscape of obesity prevalence from 1999 to 2018. In the two to five years age group, obesity rates exhibited fluctuations, experiencing a notable increase from 10.3% in 1999 to 12.5% in 2006. Subsequently, a discernible decrease of 2% was noted in 2008, reducing the prevalence to 10.5%. The following years displayed varying trends, with a decrease to 8.9% in 2014, followed by a notable increase to 13.7% in 2018, marking the highest percentage within the 1999-2018 period.

Conversely, the six to 11 years age group demonstrated significant fluctuations, ranging from 15.9% in 1999 to 17.5% in 2004. A modest decline of 0.5% prevalence occurred in 2006. From 2006 to 2010, an upward trend in obesity percentage was observed, rising from 17.0% to 18.8%, followed by a subsequent decline to 17.5% in 2014. Notably, from 2014 to 2018, there was a resurgence in obesity prevalence, reaching 19.3% in 2018, the highest within the 1999-2018 period.

**Figure 2:**
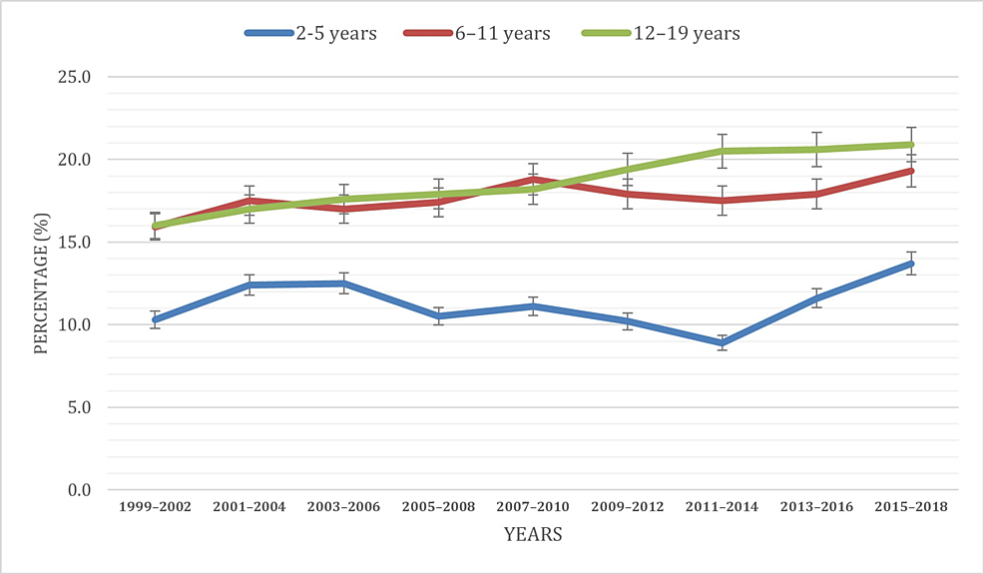
Obesity prevalence based on the age group

The 12-19 years age group exhibited a consistent rise in obesity prevalence, escalating from 16% in 1999 to 20.9% in 2018, with the highest percentage observed in the last year of the study period in 2018. These findings underscore the dynamic nature of obesity trends and emphasize the importance of tailored interventions across various developmental stages. Figure 2 visually represents the age distribution of obesity cases among individuals aged two to 19 during the study period, providing a comprehensive overview of the obesity patterns in the United States youth population.

Based on race

The investigation into obesity patterns reveals noteworthy insights when considering various racial characteristics. The analysis spans the years 1999 to 2018, offering a comprehensive understanding of how obesity prevalence varies across different racial groups. Thus, over the study period, distinctive patterns emerged within specific racial categories, revealing notable variations in obesity prevalence among children and adolescents aged two to 19. Particularly noteworthy was the highest prevalence observed among Hispanic or Latino individuals, with a prevalence rate of 22.8%. Following closely, Mexican individuals exhibited a prevalence rate of 22.0%, while Black or African American-only individuals demonstrated a similar prevalence, with a rate of 20.6%. White-only individuals displayed a lower prevalence at 14.4%, and Asian-only individuals exhibited the lowest prevalence at 9.4%.

**Figure 3:**
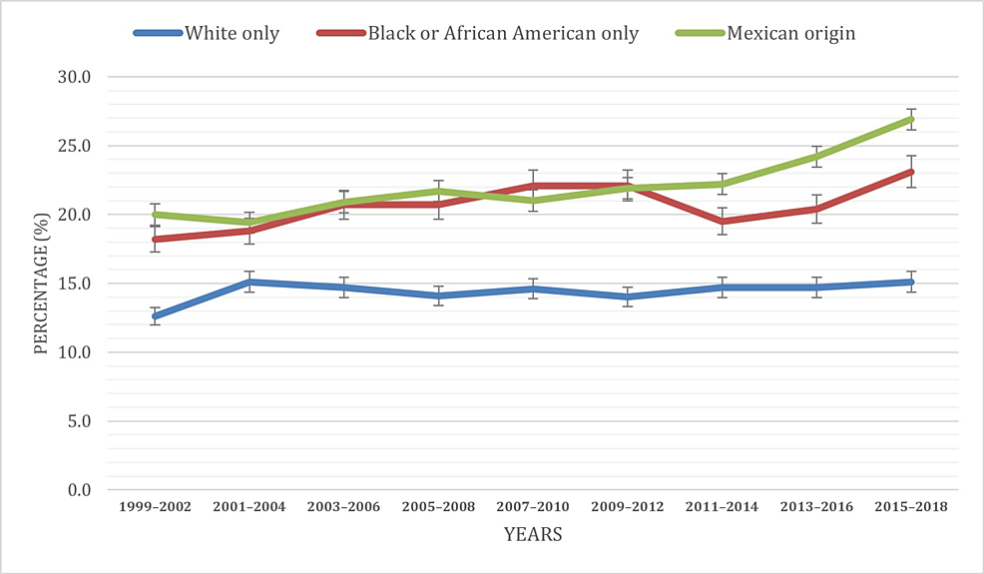
Obesity prevalence based on race

Our statistical analysis confirmed the presence of significant differences in obesity rates across these racial categories (p < 0.001). Examining temporal trends, both the Hispanic or Latino and Asian-only groups displayed an upward trajectory in obesity prevalence, reaching 25.7% and 9.4%, respectively, in 2018. Among the Black or African American population, obesity prevalence showed a generally upward trajectory from 1999 to 2018, with a notable decline from 22.1% to 19.5% observed during 2011-2014. In the case of the White-only population, obesity prevalence exhibited a non-uniform pattern, displaying fluctuations between 12.6% and 15.1% from 1999 to 2014, followed by a stagnant period at 14.7% from 2014 to 2016 and a subsequent increase to 15.1% in 2018. Notably, the Mexican-origin race category witnessed a significant 6.9% increase in prevalence from 1999 to 2018, reaching 26.9% by 2018, the highest among the observed racial categories. Figure 3 visually illustrates the distribution of obesity cases among children and adolescents aged two to 19 by race/ethnicity during the study period.

Based on the percentage of the poverty level

The analysis of obesity patterns reveals significant correlations with poverty levels. Notably, a higher prevalence of obesity is consistently observed among individuals in lower socioeconomic brackets. The data highlight that instances of obesity are more prevalent among those below 100% of the poverty level, aligning with established research findings. Throughout the study years, the below 100% poverty level accounted for approximately 20% of cases, with the 100-199%, 200-399%, and 400% or more categories contributing 18.6%, 16.6%, and 11.6%, respectively. The statistical analysis, with a p-value of <0.001, underscores the robustness of these observed differences in obesity prevalence across different poverty levels.

An upward trend in obesity prevalence is evident in two out of the four poverty level categories: below 100% and 100-199%. The below 100% category displayed an upward trend, with a slight 0.5% decline during 2011-2014. The 100-199% category exhibited a consistent increase over the study period. By contrast, the 200-399% category initially saw a rise from 14% to 17.8% from 1999 to 2004, followed by a decrease to 15.9% by 2012, and subsequently increased to 18.4% by 2018. The 400% or more category showed fluctuations, experiencing a decline from 12.6% to 11% from 1999 to 2006, followed by an increase to 11.9% by 2010, a subsequent decline to 11.4% by 2014, a rise in 2016 to 12.2%, and a subsequent decline to 10.6% in 2018.

**Figure 4:**
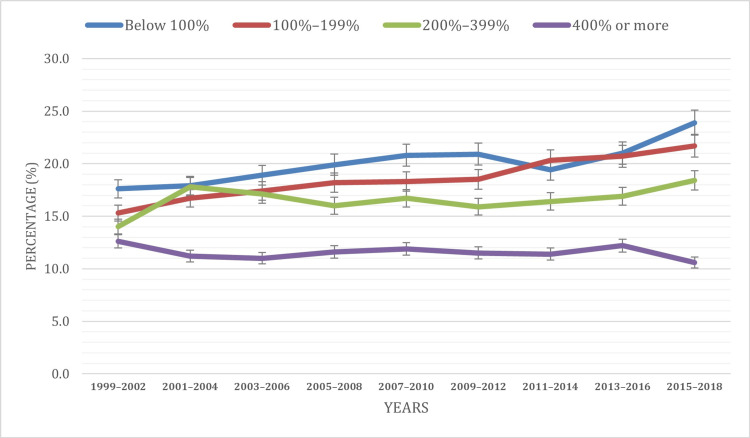
Obesity prevalence based on the percentage of poverty level

This finding underscores the complex interplay between socioeconomic factors and obesity, emphasizing the imperative for targeted interventions to address health disparities among youth from economically disadvantaged backgrounds. Figure 4 visually delineates the distribution of obesity cases among children and adolescents aged two to 19 by poverty level during the study period.

## Discussion

The comprehensive exploration of obesity patterns among children and adolescents in the United States from 1999 to 2018 has unearthed critical insights into the multifaceted nature of this public health concern. By analyzing various demographic factors, including gender, poverty level, age groups (two to five years, six to 11 years, and 12-19 years), and race/ethnicity, our study sheds light on the nuanced dynamics contributing to the prevalence of obesity within this specified population. For instance, notable gender disparities emerged, with boys consistently displaying higher prevalence rates compared to girls, mirroring established research patterns. This persistent gender gap, underscored by the study's findings and corroborated by prior research, emphasizes the imperative for a thorough investigation into the contributing factors behind this disparity. Interestingly, in the years 2011-2014, the prevalence percentage of girls slightly exceeded that of boys, indicating a temporal shift in this gender-based trend [13-15].

Distinct age-specific patterns revealed a heightened prevalence of obesity in adolescents aged 12 to 19, emphasizing unique challenges in this developmental phase of teenagers. These results align with previous research consistently noting elevated obesity rates in the broader age group of two to 19 years. The concordant patterns suggest that challenges specific to adolescence may amplify obesity prevalence, contributing to the observed higher rates during this crucial developmental stage [16-17]. The marginal 1% difference observed in the average of the six to 11 years age group compared to the 12-19 years age group indicates that the foundation of the obesity pattern is already laid during the transition from the younger to the adolescent age group.

The examination has highlighted significant race/ethnicity-based disparities, with elevated prevalence rates, particularly among children identifying as Hispanic or Latino, closely followed by Mexican-origin children. Such ethnic/racial disparities in the prevalence rates of childhood obesity have been attributed to a number of factors, including poverty, single-parent households, and neighborhood access to healthy foods and diets. Thus, poor households residing in neighborhoods lacking access to healthy diets are prone to expose their children to cheaper and unhealthy processed foods, which might lead to a high BMI in children [15-19]. Consequently, cultural aspects of different races affect their diets and uptake of physical activities, which impacts the development of childhood obesity. These findings align with prior research emphasizing consistent variations in obesity prevalence among diverse racial and ethnic groups, reaffirming the crucial need to acknowledge the nuanced impact of cultural and racial factors in shaping obesity prevalence trends [18,19].

Socioeconomic status emerges as a pivotal factor shaping obesity prevalence trends over the study's duration. Children and adolescents from low-income households consistently exhibited higher obesity prevalence rates, with those falling below the 100% poverty level contributing approximately 20% of cases. By contrast, the 400% or more poverty level category contributed the least, with an obesity prevalence of 11.6%. These findings underscore the intricate relationship between socioeconomic status and obesity, emphasizing the need for targeted interventions addressing the unique challenges faced by individuals from economically disadvantaged backgrounds [20-21].

Lastly, the notable long-term impacts of childhood obesity on public health include the observation that obesity increases the child’s risk of developing chronic and serious medical conditions, including hypertension, type 2 diabetes, orthopedic problems, liver disease, and high cholesterol [14,18]. Thus, childhood obesity reduces the life expectancy of the affected persons while also increasing the chances of chronic diseases, including auto-immune diseases that include arthritis, multiple sclerosis, cancer, and heart disease. Still, childhood obesity heightens the risk of premature mortality, even as the mortality rate has been noted to be thrice higher before attainment of 30 years of age in comparison to the general population [18-20].

Strengths and limitations of the study

The strength of this study lies in its meticulous data analysis, incorporating a wide range of variables, such as demographics, socioeconomic factors, and geographical disparities. The researchers have employed sophisticated statistical methods to provide a nuanced examination of the multifaceted nature of obesity, shedding light on potential contributing factors and identifying vulnerable populations. The utilization of a large and nationally representative dataset enhances the generalizability of the findings, making this study a pivotal resource for policymakers, healthcare professionals, and researchers alike.

While our study on obesity patterns among United States children and adolescents provides valuable insights, it is crucial to acknowledge its limitations. The retrospective nature of the study, relying on data extracted from the NCHS database spanning 1999 to 2018, imposes inherent constraints, potentially limiting the capture of recent shifts in obesity trends or evolving sociodemographic landscapes. Thus, by relying on data collected between 1999 and 2018, the most recent shifts in childhood obesity trends that might have occurred after 2018 are not taken into consideration, and this might have an impact on the application of the findings. In this regard, it is important that future qualitative studies should be conducted and should take into account the study duration by ensuring that updated and most recent data are included in the studies. The reliance on self-reported data introduces the potential for recall bias, and the categorization of race and ethnicity, while informative, may oversimplify complex identities and cultural backgrounds, potentially overlooking intra-group variations. The study predominantly relies on quantitative analysis, limiting the depth of our understanding regarding the qualitative aspects of lifestyle, cultural influences, and regional variations contributing to obesity disparities. Moreover, the scope of the study focuses on selected characteristics, excluding other influential factors, such as genetic, environmental, and behavioral aspects.

Recognizing these limitations is essential for interpreting our findings cautiously and underscores the need for complementary research methodologies to provide a more comprehensive understanding of the intricate dynamics influencing obesity patterns among children and adolescents. Moreover, based on the observed disparities between male and female children with regard to obesity prevalence rates, it is vital that future studies focus on why male children have higher rates of obesity than their female counterparts despite the males partaking in more physical activities than females.

## Conclusions

Adolescent obesity rates, particularly among those aged 12-19, are notably higher, with boys having a consistently higher prevalence than girls. Low-income households exhibit elevated rates, and Hispanic or Latino, particularly Mexican-origin, children face specific challenges. These trends emphasize the need for culturally sensitive interventions tailored to diverse populations. Addressing gender and racial/ethnic disparities through targeted interventions focusing on key determinants of weight gain is crucial. Early prevention efforts, including interventions in early childhood, group and home visits, and educating parents and children on obesity prevention, can significantly reduce childhood obesity rates.

## References

[1] Lin X, Li H (2021). Obesity: epidemiology, pathophysiology, and therapeutics. Front Endocrinol (Lausanne).

[2] Ryan DH, Kahan S (2018). Guideline recommendations for obesity management. Med Clin North Am.

[3] (2023). WHO: Obesity and overweight. https://www.who.int/news-room/fact-sheets/detail/obesity-and-overweight..

[4] (2024). CDC: About child & teen BMI. https://www.cdc.gov/healthyweight/assessing/bmi/childrens_bmi/about_childrens_bmi.html..

[5] Gee S, Chin D, Ackerson L, Woo D, Howell A (2013). Prevalence of childhood and adolescent overweight and obesity from 2003 to 2010 in an integrated health care delivery system. J Obes.

[6] (2024). CDC: childhood obesity facts. https://www.cdc.gov/obesity/data/childhood.html..

[7] Baptista LS, Silva KR, Jobeili L, Guillot L, Sigaudo-Roussel D (2023). Unraveling white adipose tissue heterogeneity and obesity by adipose stem/stromal cell biology and 3D culture models. Cells.

[8] Petersen MC, Shulman GI (2018). Mechanisms of insulin action and insulin resistance. Physiol Rev.

[9] Koh JH, Kim WU (2017). Dysregulation of gut microbiota and chronic inflammatory disease: from epithelial defense to host immunity. Exp Mol Med.

[10] Susca N, Leone P, Prete M, Cozzio S, Racanelli V (2024). Adipose failure through adipocyte overload and autoimmunity. Autoimmun Rev.

[11] (2024). CDC: Health, United States, 2020-2021. https://www.cdc.gov/nchs/hus/report.htm#citation.

[12] NHANES Questionnaires (2024). NHANES questionnaires, datasets, and related documentation. https://wwwn.cdc.gov/nchs/nhanes/.

[13] Deng Y, Yli-Piipari S, El-Shahawy O, Tamura K (2023). Trends and key disparities of obesity among US adolescents: the NHANES from 2007 to 2020. BMJ.

[14] Shah B, Tombeau Cost K, Fuller A, Birken CS, Anderson LN (2020). Sex and gender differences in childhood obesity: contributing to the research agenda. BMJ Nutr Prev Health.

[15] Nurwanti E, Hadi H, Chang JS, Chao JC, Paramashanti BA, Gittelsohn J, Bai CH (2019). Rural-urban differences in dietary behavior and obesity: results of the Riskesdas Study in 10-18-year-old Indonesian children and adolescents. Nutrients.

[16] Ogden CL, Carroll MD, Lawman HG, Fryar CD, Kruszon-Moran D, Kit BK, Flegal KM (2016). Trends in obesity prevalence among children and adolescents in the United States, 1988-1994 through 2013-2014. JAMA.

[17] Ruiz LD, Zuelch ML, Dimitratos SM, Scherr RE (2019). Adolescent obesity: diet quality, psychosocial health, and cardiometabolic risk factors. Nutrients.

[18] Hu K, Staiano AE (2022). Trends in obesity prevalence among children and adolescents aged 2 to 19 years in the US from 2011 to 2020. JAMA Pediatr.

[19] Sanyaolu A, Okorie C, Qi X, Locke J, Rehman S (2019). Childhood and adolescent obesity in the United States: a public health concern. Glob Pediatr Health.

[20] Weaver RG, Brazendale K, Hunt E, Sarzynski MA, Beets MW, White K (2019). Disparities in childhood overweight and obesity by income in the United States: an epidemiological examination using three nationally representative datasets. Int J Obes (Lond).

[21] Zare H, Gaskin DD, Thorpe RJ Jr (2021). Income inequality and obesity among US adults 1999-2016: does sex matter?. Int J Environ Res Public Health.

